# Altered brain texture features in end-stage renal disease patients: a voxel-based 3D brain texture analysis study

**DOI:** 10.3389/fnins.2024.1471286

**Published:** 2024-10-11

**Authors:** Jie Fang, Hongting Xu, Yu Zhou, Fan Zou, Jiangle Zuo, Jinmin Wu, Qi Wu, Xiangming Qi, Haibao Wang

**Affiliations:** ^1^Department of Radiology, The First Affiliated Hospital of Anhui Medical University, Hefei, China; ^2^Department of Nephrology, The First Affiliated Hospital of Anhui Medical University, Hefei, China

**Keywords:** end-stage renal disease, cognitive impairment, structural magnetic resonance imaging, brain micro-structure, texture analysis

## Abstract

**Introduction:**

Cognitive impairment in patients with end-stage renal disease (ESRD) is associated with brain structural damage. However, no prior studies have investigated the relationship between brain texture features and the cognitive function in ESRD patients. This study aimed to investigate changes in brain texture features in ESRD patients and their relationships with cognitive function using voxel-based 3D brain texture analysis (TA), and further predict individual cognitive-related brain damage in ESRD patients.

**Methods:**

Forty-seven ESRD patients and 45 control subjects underwent whole-brain high-resolution 3D T1-weighted imaging scans and neuropsychological assessments. The voxel-based 3D brain TA was performed to examine inter-group differences in brain texture features. Additionally, within the ESRD group, the relationships of altered texture features with neuropsychological function and clinical indicators were analyzed. Finally, receiver operating characteristic (ROC) curve analysis was used to evaluate the predictive ability of brain texture features for cognitive-related brain damage in ESRD patients.

**Results:**

Compared to the control group, the ESRD group exhibited altered texture features in several brain regions, including the insula, temporal lobe, striatum, cerebellum, and fusiform gyrus (*p* < 0.05, Gaussian random-field correction). Some of these altered texture features were associated with scores from the Digit Symbol Substitution Test and the Trail Making Test Parts A (*p* < 0.05), and showed significant correlations with serum creatinine and calcium levels within the ESRD group (*p* < 0.05). Notably, ROC curve analysis revealed that the texture features in the right insula and left middle temporal gyrus could accurately predict cognitive-related brain damage in ESRD patients, with the area under the curve values exceeding 0.90.

**Conclusion:**

Aberrant brain texture features may be involved in the neuropathological mechanism of cognitive decline, and have high accuracy in predicting cognitive-related brain damage in ESRD patients. TA offers a novel neuroimaging marker to explore the neuropathological mechanisms of cognitive impairment in ESRD patients, and may be a valuable tool to predict cognitive decline.

## Introduction

Chronic kidney disease is a recognized risk factor for cognitive impairment, with its prevalence increasing as kidney function declines, ranging from 30 to 62% (Viggiano et al., 2020; Kelly and Rothwell, 2022). End-stage renal disease (ESRD) is the final stage of chronic kidney disease. Cognitive decline is highly prevalent in ESRD patients, primarily affecting executive function, visuospatial function, attention, working memory, and processing speed (Drew et al., 2019; Viggiano et al., 2020). Cognitive impairment can impact patients’ quality of life, functional abilities, and treatment compliance, and is also associated with increased mortality (Drew et al., 2019; Thancharoen et al., 2020). Consequently, understanding the neuropathological basis of cognitive impairment in ESRD patients is crucial for early intervention of cognitive impairment, improving clinical management strategies, and better patient outcomes. However, the precise mechanisms responsible for cognitive decline in ESRD remain unclear.

Structural magnetic resonance imaging is a valuable tool for exploring the neural mechanisms related to cognitive impairment. Several studies have demonstrated that ESRD patients exhibit structural brain abnormalities in both brain gray and white matter, which are closely related to cognitive function. Voxel-based morphometry (Wang et al., 2022; Yuan et al., 2023; Liu et al., 2024) and surface-based morphometry (Dong et al., 2018; Gu et al., 2021; Yuan et al., 2022) methods have been used to find that the changes in cortical and subcortical structural morphology in ESRD patients, including gray matter volume, cortical thickness, and cortical complexity, which were mainly distributed in the prefrontal lobe, insula, cingulate gyrus, hippocampus, thalamus, caudate nucleus. Diffusion tensor imaging studies have revealed widespread white matter microstructure abnormalities and disruption of brain structural networks (Chou et al., 2013, 2019; Mu et al., 2018; Jiang et al., 2021), such as in the thalamic radiation, radiative crown, cingulate tract, and corpus callosum tract. Additionally, studies employing diffusion kurtosis imaging technology have detected brain micro-structural abnormalities in ESRD patients (Zheng et al., 2022, 2023). Collectively, these findings suggest that brain structural changes likely contribute to the neuropathological mechanisms of cognitive impairment in ESRD patients. However, no prior research has hitherto investigated the relationship between brain texture features and cognitive function in ESRD patients.

In recent years, texture analysis (TA) has emerged as a valuable tool for deriving imaging biomarkers. This quantitative image processing method assesses the grayscale or intensity levels of image pixels and the spatial relationships between pixels, which can capture subtle microscopic structural alterations that are invisible to the human eye, making it highly applicable in neuroimaging studies (Summers, 2017; Ghalati et al., 2022). MRI is the primary modality for TA, and its reliability has been established in previous research (Varghese et al., 2019; Ta et al., 2020). TA of MR brain structural images has become a crucial method for identifying biomarkers in various diseases, including Alzheimer’s disease (AD) (Cai et al., 2020), Parkinson’s disease (Li et al., 2019), Amyotrophic lateral sclerosis (Maani et al., 2016) and schizophrenia (Ding et al., 2024). Notably, TA holds promise for early detection of AD, predicting conversion from mild cognitive impairment to AD, and classifying AD and related disorders like mild cognitive impairment and vascular dementia (Cai et al., 2020; Luk et al., 2018; Chan et al., 2023). Furthermore, texture features are closely related to the neuropathology of AD (Lee et al., 2021). Therefore, brain TA, as an important neuroimaging marker, has the potential to provide a novel insight into the underlying neural mechanisms of cognitive impairment in ESRD patients.

The calculation of texture features is the core of TA, relying on the statistical analysis of the spatial distribution of image pixel gray values (Ghalati et al., 2022; Varghese et al., 2019). Numerous methods exist for extracting texture features. The gray co-occurrence matrix (GLCM), a fundamental and reliable TA method, is the benchmark for most TA researches, which can determine an image’s texture information by analyzing the co-occurrence frequency of adjacent pixels, reflecting the gray change relationship between them (Summers, 2017; Ghalati et al., 2022). Yang et al. (2018) investigated the dependence of texture features on MR acquisition parameters, concluding that GLCM-derived features exhibited high reliability with minimal variation (Yang et al., 2018). Maani et al. (2015) introduced the voxel-based GLCM on three orthogonal planes in 3D space (VGLCM-TOP-3D) method, enabling the extraction of texture features at the voxel level throughout the entire brain, which generates three-dimensional statistical maps of texture features, and eliminates the need for manual segmentation of regions of interest (Maani et al., 2015). This approach has been widely applied in various neurological disorders, including AD (Luk et al., 2018), schizophrenia (Ding et al., 2024), Amyotrophic Lateral Sclerosis (Maani et al., 2016), as well as medication-overuse headache (Chen et al., 2017b), demonstrating a high level of sensitivity and specificity.

In this study, the VGLCM-TOP-3D method was employed to extract texture features from whole brain 3D T1-weighted images (3D T1-WI) of ESRD and control groups. We investigated the changes in brain texture features, and their relationships with cognitive function and clinical characteristics in ESRD patients. Additionally, the receiver operating characteristic (ROC) curve analysis was used to evaluate the predictive efficacy of TA in detecting brain structural abnormalities associated with cognitive impairment in ESRD patients, thus offering a neuroimaging foundation for early detection of cognitive impairment in ESRD patients.

## Methods

### Subjects

Forty-seven ESRD patients were recruited from The First Affiliated Hospital of Anhui Medical University. These patients are undergoing peritoneal dialysis, and receive adequate dialysis with 3 to 4 times per day. The recruitment criteria for ESRD patients were as follows: glomerular filtration rate ≤ 15 mL/ (min·1.73 m^2^); aged 20–65 years; patients with stable disease without dyspnea, heart failure, nausea, fatigue, or other symptoms. Exclusion criteria were the presence of craniocerebral trauma, tumor, stroke, or other neurological diseases, history of alcohol or drug abuse, previous history of neuropsychiatric illness, central nervous medication is taken during the past 3 months or the presence of contraindications to MRI examination. We recruited 45 health control (HC) subjects, with age, gender and educational level matched, using the same exclusion criteria as that for the patients. This study was approved by the biomedical ethics committee of The First Affiliated Hospital of Anhui Medical University, and all participants provided their written informed consent to participate in this study.

### Neuropsychological assessments

All participants underwent a series of neuropsychological tests within 24 h before the MRI scan. The Mini-Mental State Examination (MMSE), Montreal Cognitive Assessment Scale (MoCA), Digit Symbol Substitution Test (DSST) and Trail Making Test Parts A (TMT-A) were performed to assess overall cognitive function, working memory, visuospatial/executive function, attention, and processing speed.

### Laboratory examinations

All patients received laboratory blood biochemical examinations within 24 h before the MR examination, including urea nitrogen, creatinine, uric acid, hemoglobin, sodium, potassium, calcium, phosphorus, ferritin, and serum iron. No blood biochemical examinations were performed in the HC group.

### MRI data acquisition

MRI data were acquired on a 3.0 T MRI system (Ingenia, Philips Healthcare, Best, Netherlands) at the Department of Radiology of The First Affiliated Hospital of Anhui Medical University. Participants remained still and stay awake with their eyes closed during the MRI scan. Conventional T2-WI were acquired to rule out subjects with significant brain structural abnormalities. The High-resolution 3D T1-WI were acquired using 3D magnetization-prepared rapid gradient-echo (MPRAGE) sequence with the following parameters: TR = 1900 ms; TE = 2.48 ms; the number of slices = 176; thickness = 1.0 mm; gap = 0 mm; matrix = 256 × 256; FA = 9°; FOV = 256 mm × 256 mm; voxel size = 1 × 1 × 1 mm^3^; scanning time = 6 min 12 s.

### Image preprocessing

Based on the MATLAB software (MATLAB R2018a, Math Works, USA), the high-dimensional DARTEL procedure in the CAT 12 toolbox^1^ was used to preprocess the 3D T1-WI data. The process involved several steps: (1) 3D T1-WI were normalized to Montreal Neurological Institute (MNI) 152 standard space, (2) segmentation of the brain into gray matter, white matter, and cerebrospinal fluid in the MNI stereotactic space with resolution of 1.5 × 1.5 × 1.5 mm^3^ voxel size, (3) noise removal and intensity standardization of the segmented image; (4) the segmented gray matter image was binarized to generate a gray matter mask using “Expression: i 1 > 0” in “ImCalc” on the SPM interface. Subsequently, these gray matter images were utilized for voxel-level texture feature extraction.

### Texture analysis

The VGLCM-TOP-3D method (Maani et al., 2015) in 3D-Texture-Analysis toolbox^2^ was used to extract gray matter texture features from the preprocessed gray matter images with 1.5 × 1.5 × 1.5 mm^3^ voxel size. This method calculates texture features in three orthogonal planes at each voxel, with the final texture feature value being the average of all three. Default parameters (neighborhood radius = 1, quantization level = 8, offset distance =1, smoothing kernel = 0) were applied for GLCM computation. Twenty-two texture features were extracted from each subject, and each texture feature subsequently mapped to the MNI152 standard space in order to generate each texture feature map.

### Statistical analysis

Demographic and neuropsychological data were analyzed using SPSS Statistics version 22.0 (SPSS 22.0). A two-sample independent *t*-test or *Chi-squared* test was performed to obtain the differences in age, educational level, neuropsychological test scores, and gender between the two groups; Statistical significance was set at *p* < 0.05.

Statistical analysis of the texture feature data was performed using the DPABI software package. A two-sample *t*-test was performed to detect differences in texture features between the two groups, with age, gender, and education level as covariates. Gaussian random field (GRF) theory was used for multiple comparisons (voxel level *p* < 0.001, cluster level *p* < 0.05, and cluster size >100 voxels).

The correlation analyses were performed by SPSS 22.0 software. Within the ESRD group, the altered texture feature values were extracted, and *Pearson* correlation coefficients were calculated to assess the relationships between these features and neuropsychological and clinical indexes (*p* < 0.05). Given the exploratory nature of these correlations, Corrections for multiple comparisons were not performed in this study.

Following the identification of texture features significantly correlated with neuropsychological scores, these features were selected as predictors for further analysis. The ROC curve analysis was used to assess the predictive efficacy of TA in detecting brain structural abnormalities related to cognitive impairment in ESRD patients (*p* < 0.05), with a 95% confidence interval (95% CI). The area under the curve (AUC) of 0.5–0.7 indicates low accuracy; 0.7–0.9 indicates average accuracy; >0.9 indicates high accuracy (Bathgate et al., 2016).

## Results

### Demographic data, neuropsychological scores, and clinical characteristics

The demographic information, neuropsychological assessment scores, and clinical indicators of the participants are displayed in Table 1. No statistically significant differences in age, gender, and educational level between the two groups (all *p* > 0.05). The MMSE, MoCA, DSST, and TMT-A scores in the ESRD group were significantly different from those in the control group (all *p* < 0.05).

**Table 1:** Demographic, cognitive and clinical data for the ESRD and HC groups.

Variables	ESRD (*n* = 47)	HC (*n* = 45)	*p*-value
Age (year)	47.02 ± 10.19	47.73 ± 10.78	0.745
Gender (male/female)	18/29	24/21	0.150
Education (years)	7.30 ± 3.06	8.44 ± 3.45	0.095
MMSE	26.23 ± 3.34	28.64 ± 1.52	<0.001
MOCA	22.89 ± 4.42	25.84 ± 2.65	<0.001
DSST	34.36 ± 16.07	41.11 ± 14.13	0.035
TMT-A	66.13 ± 34.96	50.73 ± 16.07	0.008
Disease duration (month)	84.49 ± 86.99	−	−
Urea nitrogen (mmol/L)	19.09 ± 4.55	−	−
Creatinine (μmol/L)	964.66 ± 295.57	−	−
Uric acid (μmol/L)	424.28 ± 83.05	−	−
Hemoglobin (g/L)	95.89 ± 18.74	−	−
Sodium (mmol/L)	138.57 ± 4.23	−	−
Potassium (mmol/L)	3.87 ± 0.59	−	−
Calcium (mmol/L)	2.24 ± 0.21	−	−
Phosphorus (mmol/L)	1.61 ± 0.52	−	−
Ferritin (μg/L)	308.36 ± 262.42	−	−
Serum iron (μmol/L)	13.76 ± 5.95	−	−

ESRD, end-stage renal disease; HC, health control; MMSE, Mini-Mental State Examination; MoCA, Montreal Cognitive Assessment Scale; DSST, Digit Symbol Substitution Test; TMT-A: Trail Making Test Parts A.

### Group differences of all texture features

Based on the comprehensive findings of the study, six optimal texture features (Correlation, Cluster Shade, Energy, Homogeneity 1, Inverse Difference Normalized, and Maximum Probability) were selected for further analysis. The selection was driven by the significant correlations observed between alterations in these texture features and cognitive function, as well as their high accuracy in predicting cognitive-related brain damage in the ESRD group. The description of the six texture features is shown in Supplementary Table 1 (Zwanenburg et al., 2020).

**Figure 1:**
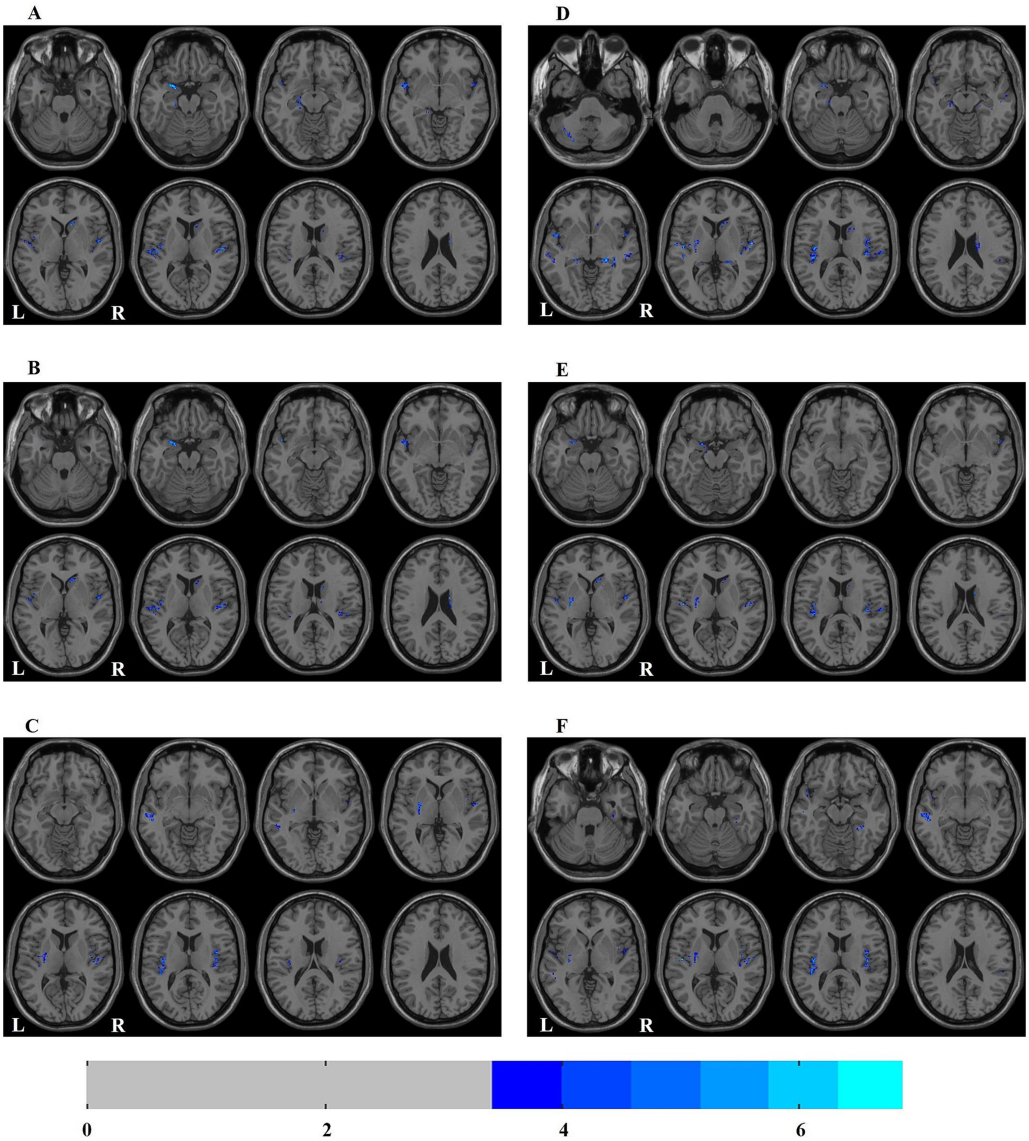
Distribution map of the brain regions with texture feature diferences between ESRD and HC groups. **(A)** correlation; **(B)** cluster shade; **(C)** energy; **(D)** homogeneity 1; **(E)** inverse difference normalized; **(F)** maximum probability. ESRD, end-stage renal disease; HC, health control; L, left; R, right.

In ESRD group, the Correlation values of bilateral superior temporal gyrus, left parahippocampal gyrus, right caudate nucleus and left amygdala were decreased (Table 2; Figure 1A); the Cluster Shade values of bilateral superior temporal gyrus, right caudate nucleus and left amygdala were decreased (Table 2; Figure 1B); the Energy values of the bilateral insula, left putamen, left cerebellum, and left middle temporal gyrus were decreased (Table 2; Figure 1C); the Homogeneity 1 values of the bilateral insula, bilateral hippocampus, bilateral middle temporal gyrus, right caudate nucleus, left cerebellum, left putamen, and left amygdala were decreased (Table 2; Figure 1D); the Inverse Difference Normalized values of the bilateral superior temporal gyrus, left putamen, and right caudate nucleus were decreased (Table 2; Figure 1E); the Maximum Probability values of bilateral insula, left middle temporal gyrus, left superior temporal gyrus, left putamen and right fusiform gyrus were decreased (Table 2; Figure 1F). No significant differences were observed in the texture features of other brain regions.

**Table 2:** Brain regions with altered texture features between the ESRD and HC group (*p* < 0.05, GRF-corrected).

Texture features	Brain regions	Voxel sizes	MNI coordinate			*T*-value
			X	Y	Z	
Correlation	Temporal_Sup_L	363	−47	−21	11	−5.97
	Temporal_Sup_R	305	53	−8	5	−5.87
	ParaHippocampal_L	123	−18	−23	−14	−5.30
	Caudate_R	115	11	13	11	−5.50
	Amygdala_L	110	−20	3	−18	−6.34
Cluster shade	Temporal_Sup_L	320	−47	−21	11	−6.09
	Temporal_Sup_R	267	59	−20	12	−5.75
	Caudate_R	164	11	14	11	−5.32
	Amygdala_L	103	−20	0	−20	−5.57
Energy	Insula_R	237	41	−3	14	−5.87
	Insula_L	187	−36	−15	15	−6.59
	Putamen_L	122	−30	−11	3	−5.70
	Cerebelum_Crus2_L	119	−35	−68	−44	−5.45
	Temporal_Mid_L	116	−53	−30	−6	−5.67
Homogeneity 1	Insula_L	523	−38	−17	15	−6.25
	Insula_R	433	36	−11	15	−5.90
	Hippocampus_R	279	23	−33	−5	−6.34
	Caudate_R	195	11	14	11	−5.37
	Hippocampus_L	138	−23	−32	−6	−5.83
	Cerebelum_Crus2_L	131	−35	−50	−48	−5.20
	Putamen_L	120	−29	−8	2	−5.51
	Temporal_Mid_L	117	−59	−23	−8	−5.34
	Temporal_Mid_R	110	62	−15	−9	−5.20
	Amygdala_L	101	−27	5	−20	−6.58
Inverse difference normalized	Temporal_Sup_R	245	53	−8	5	−5.65
	Temporal_Sup_L	223	−47	−21	11	−6.20
	Putamen_L	114	−29	−8	2	−5.52
	Caudate_R	113	11	14	11	−5.34
Maximum probability	Insula_R	304	51	−6	3	−5.60
	Insula_L	254	−36	−15	15	−6.91
	Temporal_Mid_L	134	−53	−30	−6	−6.04
	Temporal_Sup_L	127	−47	−14	8	−6.03
	Putamen_L	124	−26	−6	3	−5.40
	Fusiform_R	113	29	−45	−11	−5.29

ESRD, end-stage renal disease; HC, health control; GRF, Gaussian random field; R, right; L, left; MNI, Montreal Neurological Institute.

### Correlation analysis

In the ESRD group, DSST scores showed positive correlations with the values of texture features in the bilateral superior temporal gyrus, bilateral insula, left middle temporal gyrus, left amygdala, and right caudate nucleus (Table 3; Supplementary Figure 1). TMT-A scores showed negative correlations with the values of texture features in the left middle temporal gyrus and the right fusiform gyrus (Table 3; Supplementary Figure 2). No significant correlations were identified between altered texture features and other neuropsychological tests.

**Table 3:** Results of correlations between the altered texture features and DSST, TMT-A, urea nitrogen and serum calcium in ESRD group.

Texture features	Brain regions	DSST		TMT-A		Urea Nitrogen		Serum Calcium	
		*r*	*p*	*r*	*p*	*r*	*p*	*r*	*p*
Correlation	Temporal_Sup_L	0.334^*^	0.022	−0.146	0.327	−0.013	0.933	−0.401	0.005
	Temporal_Sup_R	0.431^**^	0.003	−0.194	0.191	0.000	0.998	−0.259	0.079
	ParaHippocampal_L	0.208	0.160	−0.187	0.209	−0.041	0.783	−0.317^*^	0.030
	Caudate_R	0.206	0.164	−0.231	0.117	−0.183	0.219	−0.095	0.527
	Amygdala_L	0.288^*^	0.049	−0.267	0.069	−0.156	0.295	−0.273	0.063
Cluster shade	Temporal_Sup_L	0.396^**^	0.006	−0.199	0.181	−0.01	0.945	−0.335^*^	0.021
	Temporal_Sup_R	0.502^**^	0.000	−0.232	0.117	0.008	0.956	−0.254	0.085
	Caudate_R	0.314^*^	0.032	−0.293^*^	0.046	−0.195	0.189	−0.110	0.461
	Amygdala_L	0.338^*^	0.020	−0.286	0.052	−0.098	0.512	−0.229	0.122
Energy	Insula_R	0.342^*^	0.019	−0.172	0.246	−0.186	0.210	−0.284	0.053
	Insula_L	0.120	0.420	−0.097	0.518	−0.300^*^	0.040	−0.337^*^	0.021
	Putamen_L	0.088	0.556	−0.018	0.904	0.162	0.278	−0.006	0.970
	Cerebelum_Crus2_L	−0.172	0.247	0.004	0.979	−0.328^*^	0.024	−0.302^*^	0.039
	Temporal_Mid_L	0.274	0.062	−0.314^*^	0.031	−0.320^*^	0.028	−0.315^*^	0.031
Homogeneity 1	Insula_L	0.323^*^	0.027	−0.177	0.235	−0.103	0.489	−0.366^*^	0.011
	Insula_R	0.442^**^	0.002	−0.209	0.159	−0.111	0.456	−0.240	0.104
	Hippocampus_R	0.272	0.065	−0.205	0.167	−0.191	0.199	−0.350^*^	0.016
	Caudate_R	0.226	0.127	−0.236	0.111	−0.222	0.134	−0.058	0.699
	Hippocampus_L	0.252	0.087	−0.239	0.106	−0.083	0.577	−0.322^*^	0.027
	Cerebelum_Crus2_L	−0.027	0.860	−0.093	0.534	−0.381^**^	0.008	−0.314^*^	0.031
	Putamen_L	0.091	0.545	−0.017	0.911	0.143	0.338	−0.019	0.900
	Temporal_Mid_L	0.309^*^	0.034	−0.188	0.207	−0.187	0.208	−0.418^**^	0.003
	Temporal_Mid_R	0.278	0.059	−0.14	0.349	−0.091	0.542	−0.418^**^	0.003
	Amygdala_L	0.258	0.080	−0.193	0.193	−0.219	0.140	−0.250	0.090
Inverse difference normalized	Temporal_Sup_R	0.442^**^	0.002	−0.221	0.135	−0.029	0.848	−0.222	0.134
	Temporal_Sup_L	0.346^*^	0.017	−0.148	0.319	−0.03	0.840	−0.358^*^	0.014
	Putamen_L	0.095	0.526	−0.020	0.893	0.137	0.360	−0.013	0.929
	Caudate_R	0.215	0.147	−0.228	0.122	−0.208	0.161	−0.058	0.698
Maximum probability	Insula_R	0.376^**^	0.009	−0.165	0.267	−0.182	0.221	−0.274	0.063
	Insula_L	0.217	0.142	−0.096	0.522	−0.234	0.113	−0.365^*^	0.012
	Temporal_Mid_L	0.284	0.053	−0.295^*^	0.044	−0.310^*^	0.034	−0.362^*^	0.013
	Temporal_Sup_L	0.261	0.077	−0.267	0.070	−0.105	0.481	−0.254	0.085
	Putamen_L	0.094	0.530	−0.016	0.917	0.144	0.333	−0.005	0.976
	Fusiform_R	0.266	0.071	−0.385^**^	0.007	−0.318^*^	0.029	−0.382^**^	0.008

ESRD, end-stage renal disease; DSST, Digit Symbol Substitution Test; TMT-A: Trail Making Test Parts A; L, left; R, right; ^*^p < 0.05; ^**^p < 0.01.

Urea nitrogen levels were negatively correlated with the texture features values of the left insula, left cerebellum, left middle temporal gyrus and right fusiform gyrus (Table 3; Supplementary Figure 3). Serum calcium levels showed negative correlations with the texture feature values of the bilateral middle temporal gyrus, bilateral hippocampus, left superior temporal gyrus, left insula, left cerebellum, and right fusiform gyrus (Table 3; Supplementary Figure 4). No significant correlations were observed between altered texture features and other clinical variables.

### ROC curve analysis

**Figure 2:**
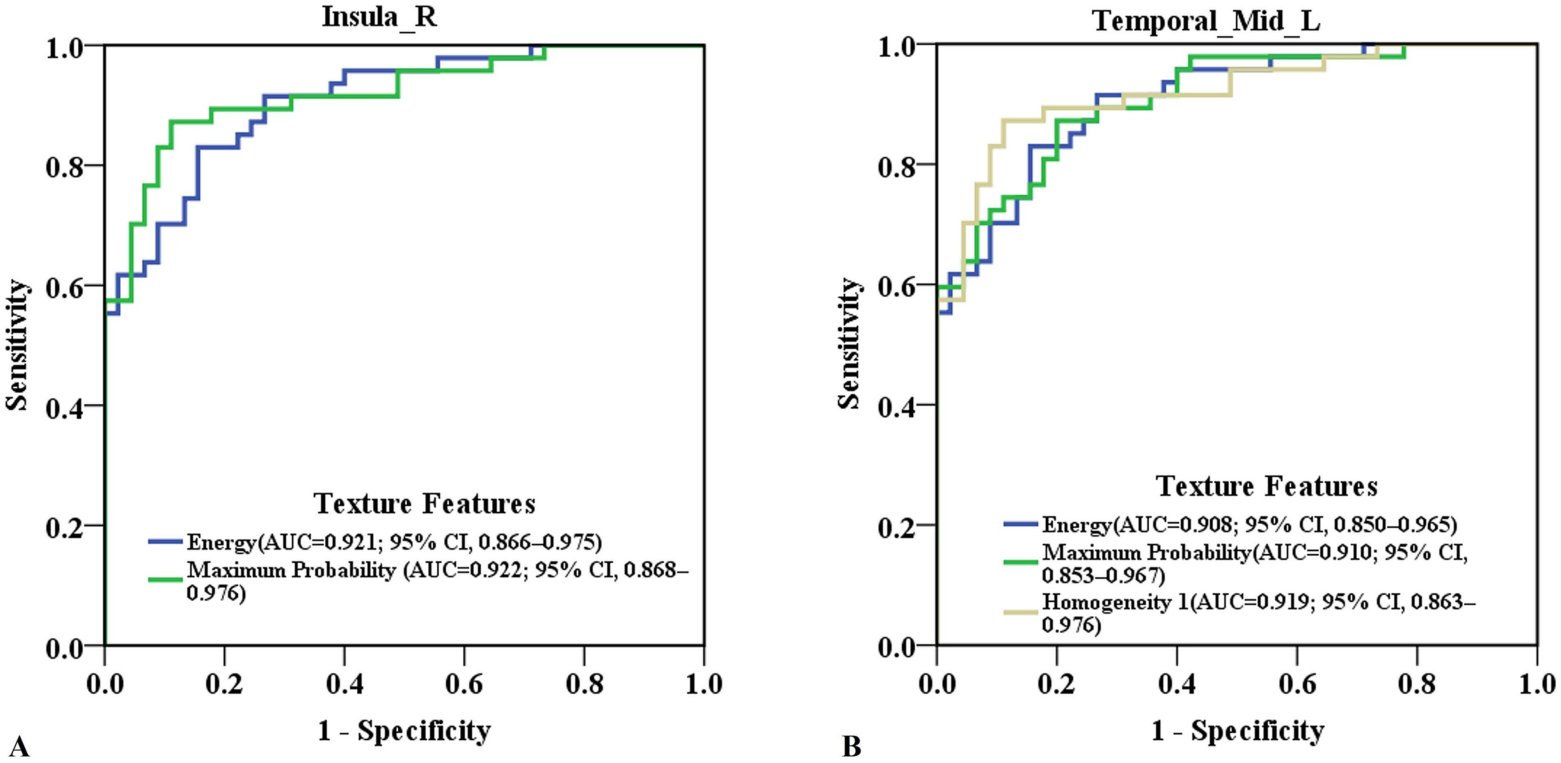
Predictive ability of texture features in detecting cognitive-related brain damage in ESRD patients. **(A)** ROC curve of energy and maximum probability in the right insula. **(B)** ROC curve of energy, homogeneity 1 and maximum probability in the left medial temporal gyrus. ESRD, end-stage renal disease; ROC, receiver operating characteristic; AUC, area under the curve, 95% CI, 95% confidence interval.

ROC curve analysis revealed that the ROC values of energy and maximum probability in the right insula were AUC (0.921; 95% CI, 0.866–0.975), accuracy (87.0%), sensitivity (89.4%), specificity (84.4%); and AUC (0.922; 95% CI, 0.868–0.976), accuracy (87.0%), sensitivity (87.2%), specificity (86.7%), respectively (Figure 2A; Supplementary Table 2). The ROC values of Energy, Homogeneity 1 and Maximum Probability in the left medial temporal gyrus were AUC (0.908; 95% CI, 0.850–0.965), accuracy (83.7%), sensitivity (83.0%), specificity (84.4%); AUC (0.919; 95% CI, 0.863–0.976), accuracy (87.0%), sensitivity (83.0%), specificity (91.1%); and AUC (0.910; 95% CI, 0.853–0.967), accuracy (83.7%), sensitivity (87.3%), specificity (80.0%), respectively (Figure 2B; Supplementary Table 2). The AUC values for texture features in other brain regions are showed in Supplementary Table 2.

## Discussion

To our knowledge, this is the first study to investigate the relationship between brain micro-structural changes and cognitive function in ESRD patients using voxel-based 3D brain TA. Our findings revealed alterations in texture features across multiple brain regions of ESRD patients. These alterations were significantly associated with cognitive function and clinical characteristics. Notably, ROC curve analysis identified that the texture features in the right insula and left middle temporal gyrus exhibited high accuracy in predicting cognitive-related brain damage in ESRD patients.

This study identified that there were several brain regions with texture abnormalities in ESRD patients, including the insula, superior temporal gyrus, middle temporal gyrus, hippocampus, parahippocampal gyrus, amygdala, caudate nucleus, lentiform nucleus, cerebellum, and fusiform gyrus. The findings demonstrated subtle alterations in the intrinsic characteristics of brain structures in ESRD patients, aligned with prior research findings (Liu et al., 2024; Zheng et al., 2022, 2023), which have confirmed altered gray matter volume and microstructure in the above brain regions. Furthermore, texture features in certain brain regions were positively correlated with the DSST test and negatively correlated with the TMT-A test. Notably, the ESRD group displayed significantly poorer performance on the MMSE, MoCA, DSST, and TMT-A than the HC group. Collectively, these findings suggest that abnormal texture features in ESRD patients may represent the neuropathological basis of cognitive decline, particularly in the domain of executive function.

Several studies have reported reduced gray matter volume and micro-structural abnormalities in the insula of ESRD patients, with these changes being closely associated with cognitive impairment (Zhang et al., 2013; Zheng et al., 2022, 2023; Yuan et al., 2023). The insula is a critical node within the salience network, playing a vital role in executive control, emotional regulation, attention, and salience processing, and is also a functional hub in switching the default mode network and the executive control network during cognitive processing (Lopez-Larson et al., 2017). In this study, the texture features of the insula were decreased and positively correlated with the DSST scores, suggesting that abnormal insula texture features may play a significant role in the neural mechanisms of impaired executive function observed in ESRD patients.

The temporal lobe is crucial for multiple cognitive functions (Vos De Wael et al., 2021). The superior and middle temporal gyrus, locating in the lateral temporal lobe, are closely related to visual perception, language processing and memory. Our study identified abnormalities in multiple texture features within these brain regions, and these features significantly correlated with the DSST and TMT-A, suggesting a potential link between abnormal texture features and impairments in visual perception and spatial memory in ESRD patients. The hippocampus, parahippocampal gyrus and amygdala locate in the medial temporal lobe. The hippocampus and parahippocampal gyrus are involved in higher cognitive functions, including memory encoding and retrieval, working memory and spatial memory (Shi et al., 2022). Previous studies on ESRD patients has documented structural damage in the hippocampus and parahippocampal gyrus, especially in those with cognitive impairment (Zheng et al., 2022; Yuan et al., 2023; Liu et al., 2024), suggesting the two brain regions paly critical roles in ESRD-related cognitive disorders. The amygdala is a key node of the emotional network, and involved in the emotion-cognition integration (Qiu et al., 2018). Given that the altered amygdala texture features were correlated with the DSST in this study, we speculate that the amygdala may involve in emotion-cognition integration in ESRD patients, which is consistent with that the interaction of emotion-cognitive function in ESRD patients in previous studies (Chen et al., 2017a; Li et al., 2018).

This study identified abnormalities in the texture features of the caudate nucleus and putamen in ESRD patients. The caudate nucleus, a crucial component of the frontostriatal circuit, is closely associated with executive function (Landau et al., 2009). Our study revealed significant correlations between caudate texture abnormalities and DSST and TMT-A, indicating a potential association between caudate abnormalities and executive dysfunction in ESRD patients. The putamen is primarily involved in the motor processing (Landau et al., 2009; Ding et al., 2018) reported reduced putamen volume, alongside a correlation between abnormal putamen functional connectivity and sensorimotor abnormalities in ESRD patients (Ding et al., 2018). Prior research has documented the atrophy and dysfunction of the caudate nucleus and putamen show correlations with various clinical factors, including dialysis factors, iron metabolism, urea levels, and hemoglobin levels (Ding et al., 2018; Gu et al., 2021; Wang et al., 2023; Zhang et al., 2024).These findings collectively suggest that the caudate nucleus and putamen are key regions of brain damage in ESRD patients, likely contributing to corresponding functional abnormalities.

We also found texture feature alterations in the cerebellum and fusiform gyrus in ESRD patients. The cerebellum, traditionally associated with motor control, has also been implicated in cognitive and emotional regulation (Chen et al., 2022). Research on cerebellar structure in ESRD patients remains limited, with few studies reporting abnormalities in cerebellar white matter microstructure (Chou et al., 2013; Jiang et al., 2021). Our finding of aberrant texture features in the cerebellar gray matter provides novel evidence of cerebellar damage in ESRD patients. Moreover, our previous study has documented abnormal functional connectivity within the cerebellum, potentially contributing to the neural mechanisms underlying cognitive impairment in ESRD (Fang et al., 2023). No significant correlation between cerebellar texture features and cognitive function was observed in the present study, which may be attributed to the heterogeneity of cerebellar involvement in cognitive decline. The fusiform gyrus known for its role in advanced visual processing, exhibited altered texture features negatively correlated with TMT-A results, suggesting that the impaired visual function of ESRD patients may be related to the abnormal fusiform texture features, which may accelerate the cognitive decline. Prior studies supported this notion, as decreased volume and functional impairment of the fusiform gyrus are more frequently observed in ESRD patients with cognitive decline (Chen et al., 2021; Yuan et al., 2023).

Brain damage in ESRD patients is associated with various clinical factors, including electrolyte imbalances, uremic toxin retention, anemia, and secondary hyperparathyroidism (Drew et al., 2019; Viggiano et al., 2020; Kelly and Rothwell, 2022). This study identified correlations between urea nitrogen and serum calcium levels and multiple brain texture features. Renal dysfunction can lead to elevated urea nitrogen, a uremic toxin, which can induce damage to the blood–brain barrier, oxidative stress, apoptosis, neurotoxicity and brain edema, ultimately result in structural and functional brain abnormalities (Hamed, 2019; Viggiano et al., 2020). Serum calcium plays an important role in the nervous system, involving in neurite atrophy, synaptic plasticity and apoptosis. Therefore, the alterations in brain texture features observed in ESRD patients may be attributed to changes in brain micro-structure resulted from the disturbances in calcium metabolism (Kong et al., 2015; Choi et al., 2017). The cumulative damage to the brain micro-structure can result in changes in the gray scale of MR images, which can be detected through TA technology (Cai et al., 2020). Previous studies have also established connections between the two factors and brain structural characteristics. These findings suggested that urea nitrogen and serum calcium are significant clinical risk factors for brain structural damage in ESRD patients (Jiang et al., 2021; Wang et al., 2022; Yuan et al., 2023). Consequently, effective management of urea nitrogen and regulation of serum calcium may offer potential avenues for alleviating brain damage in this population.

Most importantly, cognitive impairment in ESRD patients is often neglected in clinical practice. Therefore, this study used ROC curve analysis to evaluate the predictive ability of brain texture features for cognitive decline in ESRD patients. The results showed that some texture features in the insula and middle temporal gyrus exhibited AUC values exceeding 0.90. This implies that the texture features of these brain regions have high predictive accuracy for cognitive-related brain damage in ESRD patients. This finding has significant clinical implications, potentially facilitating earlier diagnosis, intervention strategies, and improved clinical management of cognitive decline in ESRD patients.

The study has several limitations. First, the sample size of patients is relatively small, we did not perform subgroup analyses. Future studies should aim to recruit larger cohorts to enable subgroup analyses. Second, hypertension, hyperlipidemia, and diabetes are common complications of ESRD. It is necessary to consider the potential influence of these factors on brain abnormalities in patients with ESRD in further studies. Third, the correction for multiple testing was not used in the correlation analyses due to the small sample size. Enlarging the sample size would allow for robust correction methods, strengthening the confidence in the observed associations. Finally, this study is a cross-sectional study. A longitudinal cohort study would be valuable for elucidating the progression of cognitive impairment and evaluating the effectiveness of potential clinical interventions in ESRD patients.

## Conclusion

In summary, this study is the first to employ voxel-based 3D brain TA to reveal abnormal brain micro-structure in ESRD patients. The altered brain texture features may be involved in the neuropathological mechanism of cognitive decline in ESRD patients. More importantly, texture features of the right insula and left middle temporal gyrus in ESRD patients have high accuracy in predicting cognitive-related brain damage. This suggests that TA may serve as a neuroimaging biomarker for investigating the neural mechanism of cognitive impairment in ESRD patients.

## Data Availability

The raw data supporting the conclusions of this article will be made available by the authors, without undue reservation.

## Footnotes

^1^ http://www.neuro.uni-jena.de/cat/

^2^ https://github.com/Pedram-Parnianpour/3D-Texture-Analysis

## References

[ref1] Bathgate C. J. Edinger J. D. Wyatt J. K. Krystal A. D. (2016). Objective but not subjective short sleep duration associated with increased risk for hypertension in individuals with insomnia. Sleep 39, 1037–1045. doi: 10.5665/sleep.5748, PMID: 26951399 PMC4835301

[ref2] Cai J.-H. He Y. Zhong X.-L. Lei H. Wang F. Luo G.-H.. (2020). Magnetic resonance texture analysis in Alzheimer’s disease. Acad. Radiol. 27, 1774–1783. doi: 10.1016/j.acra.2020.01.006 32057617

[ref3] Chan K. Fischer C. Maralani P. J. Black S. E. Moody A. R. Khademi A. (2023). Alzheimer’s and vascular disease classification using regional texture biomarkers in FLAIR MRI. NeuroImage Clin. 38:103385. doi: 10.1016/j.nicl.2023.103385, PMID: 36989851 PMC10074987

[ref4] Chen H. J. Wang Y. F. Qi R. Schoepf U. J. Varga-Szemes A. Ball B. D.. (2017a). Altered amygdala resting-state functional connectivity in maintenance hemodialysis end-stage renal disease patients with depressive mood. Mol. Neurobiol. 54, 2223–2233. doi: 10.1007/s12035-016-9811-8, PMID: 26941102

[ref5] Chen P. Hu R. Gao L. Wu B. Peng M. Jiang Q.. (2021). Abnormal degree centrality in end-stage renal disease (ESRD) patients with cognitive impairment: a resting-state functional MRI study. Brain Imaging Behav. 15, 1170–1180. doi: 10.1007/s11682-020-00317-3, PMID: 32902798

[ref6] Chen Z. Chen X. Chen Z. Liu M. He H. Ma L.. (2017b). Alteration of gray matter texture features over the whole brain in medication-overuse headache using a 3-dimentional texture analysis. J. Headache Pain 18:112. doi: 10.1186/s10194-017-0820-4, PMID: 29285575 PMC5745370

[ref7] Chen Z. Zhang R. Huo H. Liu P. Zhang C. Feng T. (2022). Functional connectome of human cerebellum. NeuroImage 251:119015. doi: 10.1016/j.neuroimage.2022.119015 35189360

[ref8] Choi H. Kim H. J. Kim J. Kim S. Yang J. Lee W.. (2017). Increased acetylation of Peroxiredoxin1 by HDAC6 inhibition leads to recovery of Aβ-induced impaired axonal transport. Mol. Neurodegener. 12:23. doi: 10.1186/s13024-017-0164-1, PMID: 28241840 PMC5330132

[ref9] Chou M.-C. Hsieh T.-J. Lin Y.-L. Hsieh Y.-T. Li W.-Z. Chang J.-M.. (2013). Widespread white matter alterations in patients with end-stage renal disease: a Voxelwise diffusion tensor imaging study. Am. J. Neuroradiol. 34, 1945–1951. doi: 10.3174/ajnr.A3511, PMID: 23788598 PMC7965420

[ref10] Chou M.-C. Ko C.-H. Chang J.-M. Hsieh T.-J. (2019). Disruptions of brain structural network in end-stage renal disease patients with long-term hemodialysis and normal-appearing brain tissues. J. Neuroradiol. 46, 256–262. doi: 10.1016/j.neurad.2018.04.004, PMID: 29733919

[ref11] Ding D. Li P. Ma X. Dun W. Yang S. Ma S.. (2018). The relationship between putamen-SMA functional connectivity and sensorimotor abnormality in ESRD patients. Brain Imaging Behav. 12, 1346–1354. doi: 10.1007/s11682-017-9808-6, PMID: 29234958

[ref12] Ding H. Zhang Y. Xie Y. Du X. Ji Y. Lin L.. (2024). Individualized texture similarity network in schizophrenia. Biol. Psychiatry 96, 176–187. doi: 10.1016/j.biopsych.2023.12.025, PMID: 38218309

[ref13] Dong J. Ma X. Lin W. Liu M. Fu S. Yang L.. (2018). Aberrant cortical thickness in neurologically asymptomatic patients with end-stage renal disease. Neuropsychiatr. Dis. Treat. 14, 1929–1939. doi: 10.2147/NDT.S170106, PMID: 30122925 PMC6080870

[ref14] Drew D. A. Weiner D. E. Sarnak M. J. (2019). Cognitive impairment in CKD: pathophysiology, management, and prevention. Am. J. Kidney Dis. 74, 782–790. doi: 10.1053/j.ajkd.2019.05.017, PMID: 31378643 PMC7038648

[ref15] Fang J. Miao Y. Zou F. Liu Y. Zuo J. Qi X.. (2023). Altered resting-state cerebellar-cerebral functional connectivity in patients with end-stage renal disease. Ren. Fail. 45:2238829. doi: 10.1080/0886022X.2023.2238829, PMID: 37488933 PMC10392254

[ref16] Ghalati M. K. Nunes A. Ferreira H. Serranho P. Bernardes R. (2022). Texture analysis and its applications in biomedical imaging: a survey. IEEE Rev. Biomed. Eng. 15, 222–246. doi: 10.1109/RBME.2021.3115703, PMID: 34570709

[ref17] Gu W. He R. Su H. Ren Z. Zhang L. Yuan H.. (2021). Changes in the shape and volume of subcortical structures in patients with end-stage renal disease. Front. Hum. Neurosci. 15:778807. doi: 10.3389/fnhum.2021.778807, PMID: 34975435 PMC8716492

[ref18] Hamed S. A. (2019). Neurologic conditions and disorders of uremic syndrome of chronic kidney disease: presentations, causes, and treatment strategies. Expert. Rev. Clin. Pharmacol. 12, 61–90. doi: 10.1080/17512433.2019.1555468, PMID: 30501441

[ref19] Jiang Y. Gao Q. Liu Y. Gao B. Che Y. Lin L.. (2021). Reduced white matter integrity in patients with end-stage and non-end-stage chronic kidney disease: a tract-based spatial statistics study. Front. Hum. Neurosci. 15:774236. doi: 10.3389/fnhum.2021.774236, PMID: 34955791 PMC8709581

[ref20] Kelly D. M. Rothwell P. M. (2022). Disentangling the relationship between chronic kidney disease and cognitive disorders. Front. Neurol. 13:830064. doi: 10.3389/fneur.2022.830064, PMID: 35280286 PMC8914950

[ref21] Kong W. Mou X. Zhang N. Zeng W. Li S. Yang Y. (2015). The construction of common and specific significance subnetworks of Alzheimer’s disease from multiple brain regions. Biomed Res. Int. 2015, 1–13. doi: 10.1155/2015/394260, PMID: 25866779 PMC4383160

[ref22] Landau S. M. Lal R. O’Neil J. P. Baker S. Jagust W. J. (2009). Striatal dopamine and working memory. Cereb. Cortex 19, 445–454. doi: 10.1093/cercor/bhn095, PMID: 18550595 PMC2733326

[ref23] Lee S. Kim K. W. (2021). Associations between texture of T1-weighted magnetic resonance imaging and radiographic pathologies in Alzheimer’s disease. Eur. J. Neurol. 28, 735–744. doi: 10.1111/ene.14609, PMID: 33098172

[ref24] Li A. Mu J. Huang M. Zhang Z. Liu J. Zhang M. (2018). Altered amygdala-related structural covariance and resting-state functional connectivity in end-stage renal disease patients. Metab. Brain Dis. 33, 1471–1481. doi: 10.1007/s11011-018-0254-y 29869149

[ref25] Li G. Zhai G. Zhao X. An H. Spincemaille P. Gillen K. M.. (2019). 3D texture analyses within the substantia nigra of Parkinson’s disease patients on quantitative susceptibility maps and R2∗ maps. NeuroImage 188, 465–472. doi: 10.1016/j.neuroimage.2018.12.041, PMID: 30578927

[ref26] Liu Y. Wang H. Sha G. Cao Y. Chen Y. Chen Y.. (2024). The covariant structural and functional neuro-correlates of cognitive impairments in patients with end-stage renal diseases. Front. Neurosci. 18:1374948. doi: 10.3389/fnins.2024.1374948, PMID: 38686326 PMC11056510

[ref27] Lopez-Larson M. P. Shah L. M. Weeks H. R. King J. B. Mallik A. K. Yurgelun-Todd D. A.. (2017). Abnormal functional connectivity between default and salience networks in pediatric bipolar disorder. Biol. *Psychiatry. Cogn. Neurosci. Neuroimaging.* 2, 85–93. doi: 10.1016/j.bpsc.2016.10.001, PMID: 29560889 PMC6422527

[ref28] Luk C. C. Ishaque A. Khan M. Ta D. Chenji S. Yang Y.. (2018). Alzheimer’s disease: 3-dimensional MRI texture for prediction of conversion from mild cognitive impairment. Alz. Dem. Diag. Ass. Dis. Mo. 10, 755–763. doi: 10.1016/j.dadm.2018.09.002, PMID: 30480081 PMC6240791

[ref29] Maani R. Yang Y. H. Kalra S. (2015). Voxel-based texture analysis of the brain. PLoS One 10:e0117759. doi: 10.1371/journal.pone.0117759, PMID: 25756621 PMC4355627

[ref30] Maani R. Yang Y.-H. Emery D. Kalra S. (2016). Cerebral degeneration in amyotrophic lateral sclerosis revealed by 3-dimensional texture analysis. Front. Neurosci. 10:120. doi: 10.3389/fnins.2016.00120, PMID: 27064416 PMC4811946

[ref31] Mu J. Chen T. Li P. Ding D. Ma X. Zhang M.. (2018). Altered white matter microstructure mediates the relationship between hemoglobin levels and cognitive control deficits in end-stage renal disease patients. Hum. Brain Mapp. 39, 4766–4775. doi: 10.1002/hbm.24321 30062855 PMC6866371

[ref32] Qiu L. Xia M. Cheng B. Yuan L. Kuang W. Bi F.. (2018). Abnormal dynamic functional connectivity of amygdalar subregions in untreated patients with first-episode major depressive disorder. J. Psychiatry Neurosci. 43, 262–272. doi: 10.1503/jpn.170112 29947609 PMC6019355

[ref33] Shi Y.-D. Ge Q.-M. Lin Q. Liang R.-B. Li Q.-Y. Shi W.-Q.. (2022). Functional connectivity density alterations in children with strabismus and amblyopia based on resting-state functional magnetic resonance imaging (fMRI). BMC Ophthalmol. 22:49. doi: 10.1186/s12886-021-02228-3, PMID: 35109804 PMC8808980

[ref34] Summers R. M. (2017). Texture analysis in radiology: does the emperor have no clothes? Abdom. Radiol. 42, 342–345. doi: 10.1007/s00261-016-0950-1, PMID: 27770161

[ref35] Ta D. Khan M. Ishaque A. Seres P. Eurich D. Yang Y.. (2020). Reliability of 3D texture analysis: a multicenter MRI study of the brain. Magn. Reson. Med. 51, 1200–1209. doi: 10.1002/jmri.26904, PMID: 31423714

[ref36] Thancharoen O. Waleekhachonloet O. Limwattananon C. Anutrakulchai S. (2020). Cognitive impairment, quality of life and healthcare utilization in patients with chronic kidney disease stages 3 to 5. Nephrology 25, 625–633. doi: 10.1111/nep.13705, PMID: 32133699

[ref37] Varghese B. A. Cen S. Y. Hwang D. H. Duddalwar V. A. (2019). Texture analysis of imaging: what radiologists need to know. Am. J. Roentgenol. 212, 520–528. doi: 10.2214/AJR.18.20624, PMID: 30645163

[ref38] Viggiano D. Wagner C. A. Martino G. Nedergaard M. Zoccali C. Unwin R.. (2020). Mechanisms of cognitive dysfunction in CKD. Nat. Rev. Nephrol. 16, 452–469. doi: 10.1038/s41581-020-0266-9 32235904

[ref39] Vos De Wael R. Royer J. Tavakol S. Wang Y. Paquola C. Benkarim O.. (2021). Structural connectivity gradients of the temporal lobe serve as multiscale axes of brain organization and cortical evolution. Cereb. Cortex 31, 5151–5164. doi: 10.1093/cercor/bhab149, PMID: 34148082 PMC8491677

[ref40] Wang H. Huang L. Wu G. Li J. Liu L. Zhang T.. (2022). Regional cerebral gray matter atrophy is associated with cognitive impairment in hemodialysis patients: a cross-sectional and longitudinal voxel-based morphological MRI study. Brai. Imaging. Behav. 16, 1284–1293. doi: 10.1007/s11682-021-00602-9, PMID: 34993881

[ref41] Wang H. Song L. Li M. Yang Z. Wang Z.-C. (2023). Association between susceptibility value and cerebral blood flow in the bilateral putamen in patients undergoing hemodialysis. J. Cereb. Blood Flow Metab. 43, 433–445. doi: 10.1177/0271678X221134384, PMID: 36284493 PMC9941863

[ref42] Yang F. Dogan N. Stoyanova R. Ford J. C. (2018). Evaluation of radiomic texture feature error due to MRI acquisition and reconstruction: a simulation study utilizing ground truth. Phys. Med. 50, 26–36. doi: 10.1016/j.ejmp.2018.05.017, PMID: 29891091

[ref43] Yuan H. Li H. Mu J. Gu W. Zhu X. Gao L.. (2022). Reduced cortical complexity in patients with end-stage kidney disease prior to dialysis initiation. Front. Neurosci. 16:971010. doi: 10.3389/fnins.2022.971010, PMID: 36389216 PMC9659747

[ref44] Yuan H. Luo Z. Gu W. Ma S. Li G. Ding D.. (2023). Abnormal grey matter structural changes in patients with end-stage kidney disease and mild cognitive impairment: correlations with clinical features. Metab. Brain Dis. 38, 2817–2829. doi: 10.1007/s11011-023-01293-5, PMID: 37776380 PMC10663233

[ref45] Zhang C. Cai Y. Yu H. Wu N. Liu J. Liang S.. (2024). Comparison of the effects of peritoneal dialysis and hemodialysis on spontaneous brain activity in CKD patients: an rs-fMRI study. Cereb. Cortex 34:bhad377. doi: 10.1093/cercor/bhad377, PMID: 37948670

[ref46] Zhang L. J. Wen J. Ni L. Zhong J. Liang X. Zheng G.. (2013). Predominant gray matter volume loss in patients with end-stage renal disease: a voxel-based morphometry study. Metab. Brain Dis. 28, 647–654. doi: 10.1007/s11011-013-9438-7, PMID: 24065440

[ref47] Zheng J. Jiao Z. Dai J. Liu T. Shi H. (2022). Abnormal cerebral micro-structures in end-stage renal disease patients related to mild cognitive impairment. Eur. J. Radiol. 157:110597. doi: 10.1016/j.ejrad.2022.110597, PMID: 36379097

[ref48] Zheng J. Sun Q. Wu X. Dou W. Pan J. Jiao Z.. (2023). Brain Micro-structural and functional alterations for cognitive function prediction in the end-stage renal disease patients undergoing maintenance hemodialysis. Acad. Radiol. 30, 1047–1055. doi: 10.1016/j.acra.2022.06.019, PMID: 35879210

[ref49] Zwanenburg A. Leger S. Vallières M. Löck S. (2020). Image biomarker standardisation initiative. Radiology 295, 328–338. doi: 10.1148/radiol.2020191145, PMID: 32154773 PMC7193906

