# Supplementary material for: Altered brain texture features in end-stage renal disease patients: a voxel-based 3D brain texture analysis study
Source: Front Neurosci. 2024 Oct 11;18:1471286. doi: 10.3389/fnins.2024.1471286 (PMC11502495; doi:10.3389/fnins.2024.1471286)
Supplement: Supplementary file 1 [file Data_Sheet_1.docx]

**Altered Brain Texture Features in End-Stage Renal Disease Patients : A Voxel-Based 3D Brain Texture Analysis Study**

**Supplementary Table 1**

Equation and description of selected texture features.

| Texture features | Equation | Description |
| --- | --- | --- |
| Correlation | $\frac{1}{\sigma i.\sigma.j}(\sum_{i=1}^{N_{g}} \sum_{j=1}^{N_{g}} ijpij-\mu i.\mu.j)$ | Correlation show the linear dependency of gray level values to their respective voxels in the GLCM. |
| Cluster Shade | $\sum_{i=1}^{N_{g}} \sum_{j=1}^{N_{g}} (i+j-\mu i. - \mu.j )^{3}pij$ | Cluster Shade is a measure of the skewness and uniformity of the GLCM. A higher cluster shade implies greater asymmetry about the mean. |
| Energy | $\sum_{i=1}^{N_{g}} \sum_{j=1}^{N_{g}} p{}^{2}ij$ | Energy is a measure of homogeneous patterns in the image. It reflects the uniformity of image gray distribution and texture fineness. A greater Energy implies that there are more instances of intensity value pairs in the image that neighbor each other at higher frequencies. |
| Homogeneity 1 | $\sum_{i=1}^{N_{g}} \sum_{j=1}^{N_{g}} \frac{pij}{1+\vert i-j\vert}$ | Homogeneity 1 is one measure of the local homogeneity of an image. |
| Inverse Difference Normalized | $\sum_{i=1}^{N_{g}} \sum_{j=1}^{N_{g}} \frac{pij}{1+\vert i-j\vert/N_{g}}$ | Inverse difference normalized is another measure of the local homogeneity of an image. Unlike Homogeneity1, Inverse difference normalized normalizes the difference between the neighboring intensity values by dividing over the total number of discrete intensity values. |
| Maximum Probability | $\max(pij)$ | Maximum Probability is occurrences of the most predominant pair of neighboring intensity values. |

**Supplementary Table 2**

Statistical parameters of the ROC curve analysis.

| Texture features | Brain regions^#^ | AUC | 95%CI | | accuracy | sensitivity | specificity |
| --- | --- | --- | --- | --- | --- | --- | --- |
|  |  |  | Lower Bound | Upper Bound |  |  |  |
| Correlation | Temporal_Sup_L | 0.863 | 0.791 | 0.936 | − | − | − |
|  | Temporal_Sup_R | 0.885 | 0.816 | 0.955 | − | − | − |
|  | Amygdala_L | 0.847 | 0.770 | 0.923 | − | − | − |
| Cluster Shade | Temporal_Sup_L | 0.821 | 0.738 | 0.905 | − | − | − |
|  | Temporal_Sup_R | 0.832 | 0.749 | 0.914 | − | − | − |
|  | Caudate_R | 0.775 | 0.679 | 0.871 | − | − | − |
|  | Amygdala_L | 0.811 | 0.722 | 0.900 | − | − | − |
| Energy | Insula_R | 0.921 | 0.866 | 0.975 | 87.0% | 89.4% | 84.4% |
|  | Temporal_Mid_L | 0.908 | 0.850 | 0.965 | 83.7% | 83.0% | 84.4% |
| Homogeneity 1 | Insula_L | 0.889 | 0.826 | 0.953 | − | − | − |
|  | Insula_R | 0.891 | 0.824 | 0.958 | − | − | − |
|  | Temporal_Mid_L | 0.919 | 0.863 | 0.976 | 87.0% | 83.0% | 91.1% |
| Inverse Difference Normalized | Temporal_Sup_R | 0.878 | 0.806 | 0.949 | − | − | − |
|  | Temporal_Sup_L | 0.865 | 0.794 | 0.936 | − | − | − |
| Maximum Probability | Insula_R | 0.922 | 0.868 | 0.976 | 87.0% | 87.2% | 86.7% |
|  | Temporal_Mid_L | 0.910 | 0.853 | 0.967 | 83.7% | 87.3% | 80.0% |
|  | Fusiform_R | 0.884 | 0.815 | 0.953 | − | − | − |

ROC, receiver operating characteristic; AUC, area under the curve; 95%CI, 95% confidence interval.

^#^, Altered texture features values of these brain regions showed correlations with neuropsychological assessments scores.

−, The accuracy, sensitivity and specificity were not reported, due to the AUC values of these brain region are lower than 0.9.

**
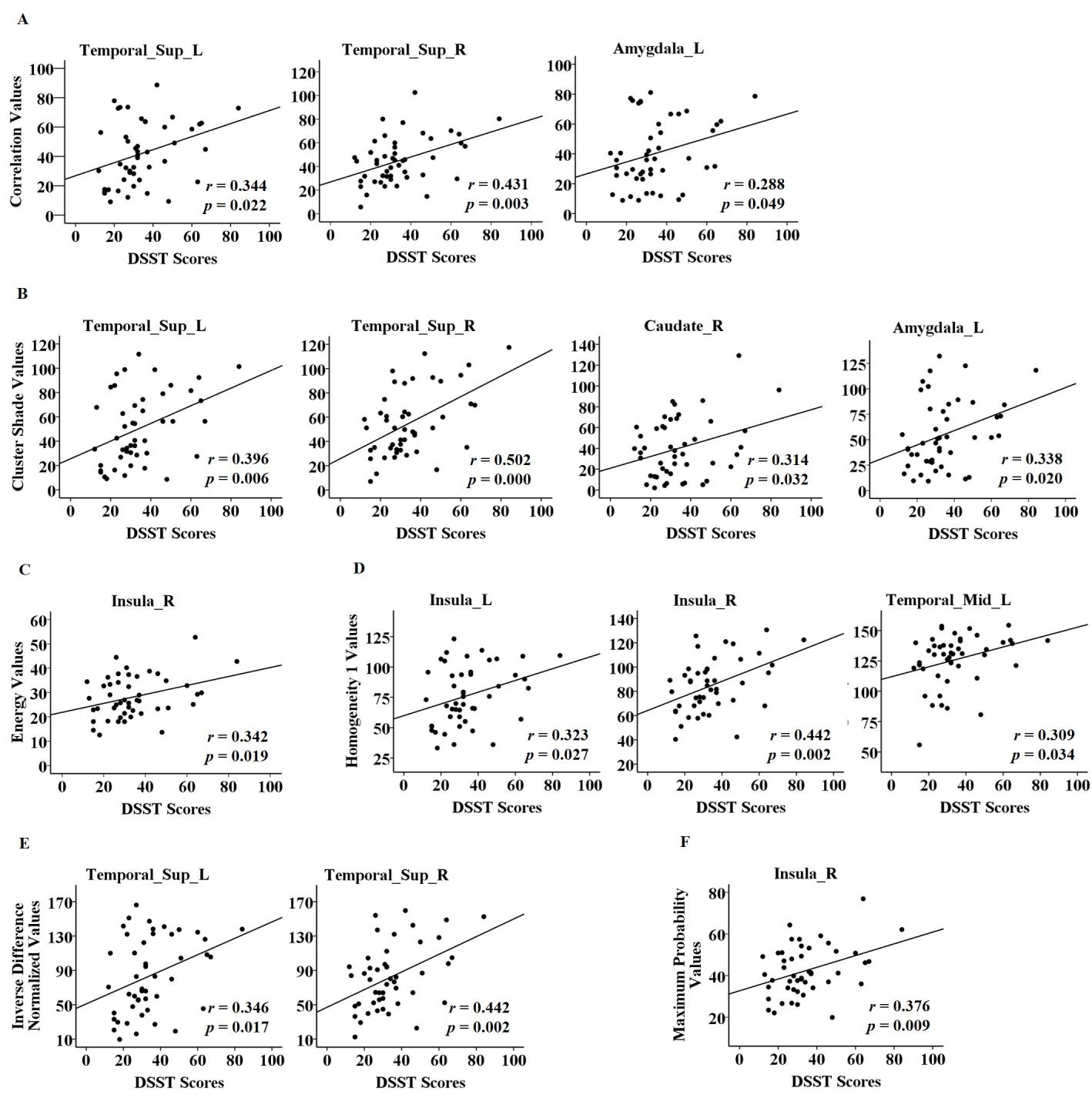
**

**Supplementary Figure 1.** Scatter plot of altered texture features values with DSST scores in the ESRD group. ESRD, end-stage renal disease; DSST, Digit Symbol Substitution Test; L, left; R, right.


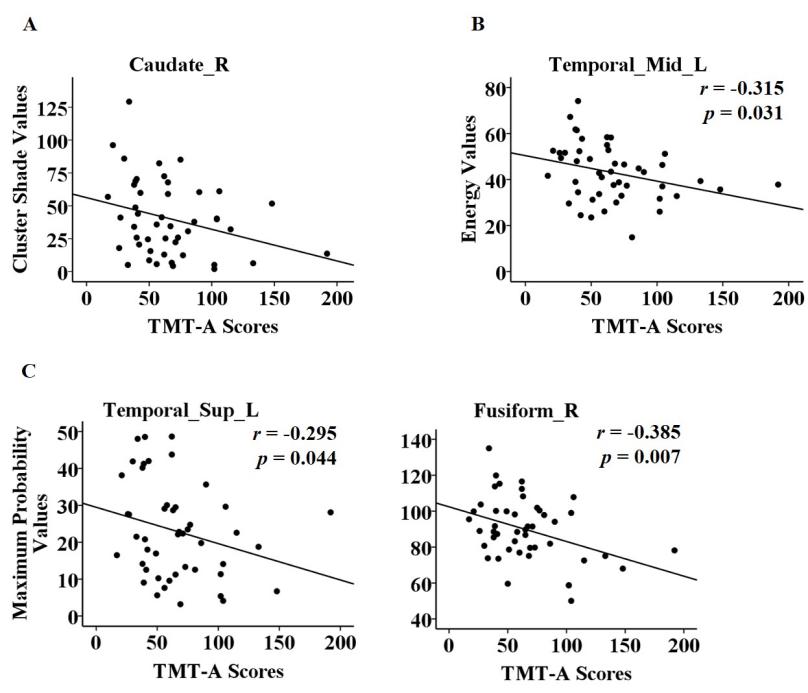


**Supplementary Figure 2.** Scatter plot of altered texture features values with TMT-A scores in the ESRD group. ESRD, end-stage renal disease; TMT-A: Trail Making Test Parts A; L, left; R, right.


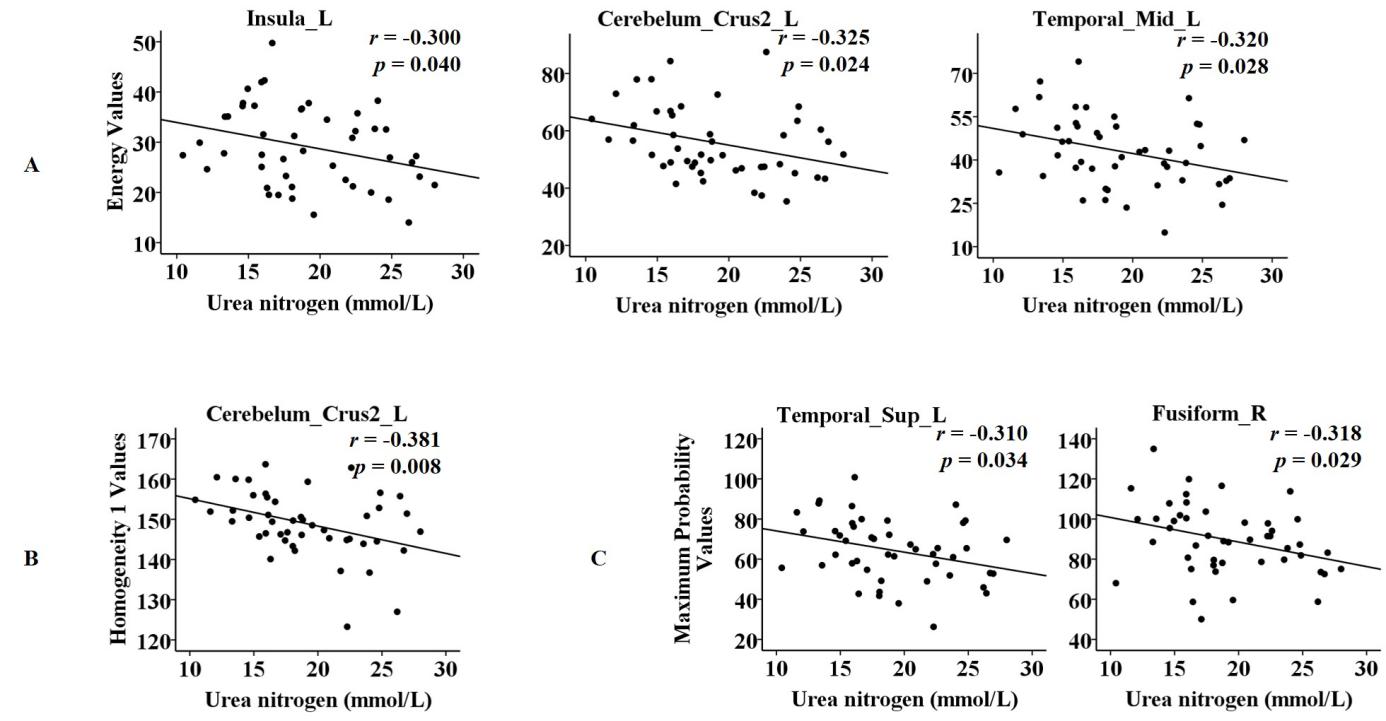


**Supplementary Figure 3.** Scatter plot of altered texture features values with urea nitrogen in the ESRD group. ESRD, end-stage renal disease; L, left; R, right.

**
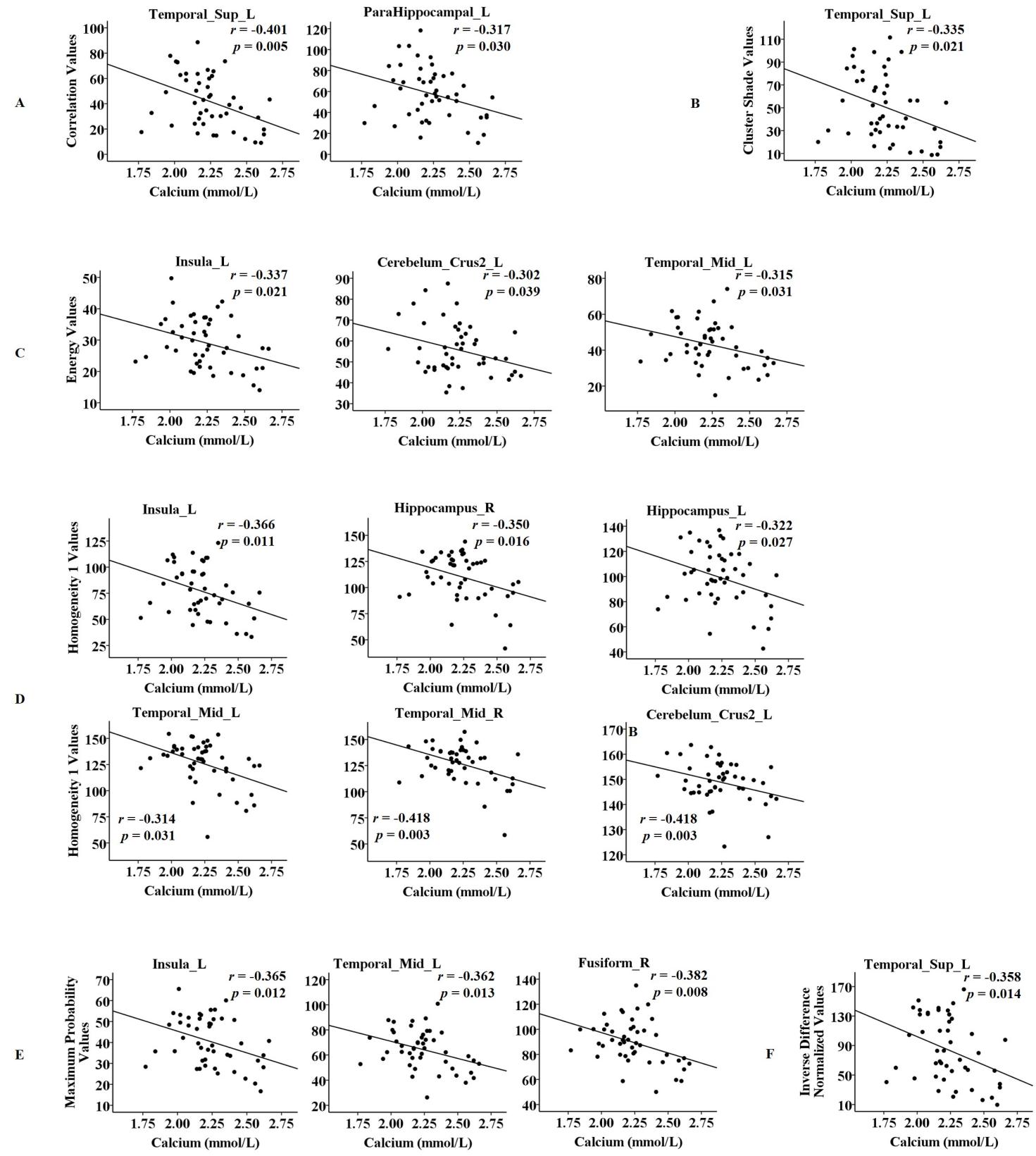
**

**Supplementary Figure 4.** Scatter plot of altered texture features values with serum calcium in the ESRD group. ESRD, end-stage renal disease; L, left; R, right.
